# The transcription factor Ap-1 regulates monkey 20α-hydroxysteroid dehydrogenase promoter activity in CHO cells

**DOI:** 10.1186/1472-6750-14-71

**Published:** 2014-07-30

**Authors:** Tsevelmaa Nanjidsuren, Kwan-Sik Min

**Affiliations:** 1 Animal Biotechnology, Graduate School of Bio and Information Technology, Institute of Genetic Engineering, Hankyong National University, Ansung 456-749, Republic of Korea

**Keywords:** Macaque monkey, 20α-HSD, Transcription factor, Ap-1, Sp-1, CHO-K1 cells

## Abstract

**Background:**

Monkey 20α-hydroxysteroid dehydrogenase (20α-HSD) is a catabolic enzyme responsible for converting progesterone into biologically inactive 20α-hydroxyprogesterone, thereby playing a key role in the estrous cycle or pregnancy and allowing ovulation and parturition to occur in most mammalian animals. Monkey 20α-HSD was highly abundant in ovarian and placental tissues during the pre-ovulation and pre-parturition phase and was primarily localized in the syncytiotrophoblast of the placenta. In this study, we focused on the molecular characterization of the monkey *20α-HSD* promoter region by conducting reporter assays in Chinese hamster ovary (CHO) K1 cells.

**Results:**

A reporter assay using constructs of various lengths of the 5′-flanking region (-890-Luc, -513-Luc, -306-Luc, -273-Luc, and -70-Luc) revealed that a region corresponding to the activator protein 1 (Ap-1) located between -281 and -274 bp was essential for the transcriptional activity. Absence of the Ap-1 site in -273-Luc dramatically decreased the transcription levels to the control levels. When the reporter constructs were co-transfected with Ap-1 (Jun) and specificity protein (Sp-1) genes, the transcription activities of the constructs increased with the exception of -273 and -70, while that of the double construct was reduced compared to that of Ap-1 alone. Furthermore, mutational analysis demonstrated that a putative *Ap-1* site played an important role in the expression of the reporter gene. These findings were confirmed by EMSA examining the interactions of the protein Ap-1 in a nuclear extract from CHO-K1 cells and the expression levels of the Ap-1 transcription factor in pre-parturition placenta and CHO-K1 cells. Although mut-1 and mut-2 of Ap-1 bound with nuclear extracts from CHO-K1 cells, the transcriptional activity of mut-3 was almost completely suppressed.

**Conclusions:**

Our results indicate that the Ap-1 site (-281 → -274) (5′-TGTCTCAT-3′) plays a crucial role in the activation of the monkey *20α-HSD* gene. Thus, we demonstrated that monkey *20α-HSD* promoter activity is regulated by the transcription factor Ap-1 in CHO-K1 cells.

## Background

The steroid-metabolizing enzyme 20α-hydroxysteroid dehydrogenase (20α-HSD; EC.1.1.1.149) catalyzes the conversion of progesterone, a potent progestin, to the metabolically inactive form 20α-dihydroprogesterone (20α-OHP) [1]. This enzyme belongs to the aldo-keto reductase (AKR) superfamily of nicotinamide adenine dinucleotide phosphate (NADPH)-dependent reductases that act on a wide range of substrates, including steroid hormones and endogenous prostaglandins [2,3]. 20α-HSD plays a crucial role in the modulation and regulation of steroid hormones, such as androgen, estrogen, and progestins [4].

The activity of 20α-HSD is known to be down-regulated by prolactin (PRL) [5], allowing for the maintenance of progesterone secretion during the first half of pregnancy. However, PGF_2_α stimulates the expression of 20α-HSD at the end of the pregnancy [6,7]. Administration of PGF_2_α to pregnant rats increases luteal 20α-HSD activity [8]. PGF_2_α is also known to induce abortion in many species, including rodents [9]. PGF_2_α receptor-deficient mice do not show the normal pre-partum drop in progesterone [10,11]. Placental lactogen (PL) is another suppressor of *20α-HSD* gene expression. The luteotrophic effects of both PRL and PL on the corpus luteum (CL) are mediated via the PRL receptor [12]. Progesterone production in the rodent CL is regulated by hormones, including PRL and PGF_2_α, which have luteotropic and luteolytic functions, respectively [7]. There is additional evidence that PGF_2_α may act as an inhibitor of the lactogenic suppression of 20α-HSD expression by increasing the expression of a member of the suppressor of cytokine signaling (SOCS) family, SOCS-3, which has been demonstrated to interfere with cytokine signaling through the Jak2/Stat5 pathway [13].

20α-HSD-deficient mice sustain high progesterone levels and present a delay in parturition of several days, demonstrating that 20α-HSD regulates parturition downstream of the PGF_2_α receptor in an essential and nonredundant manner. 20α-HSD deficiency partially corrected the abortion of pregnancies associated with Stab5b deficiency [14]. The duration of the estrous cycle, pseudopregnancy, and pregnancy was significantly prolonged in 20α-HSD ^-^/^-^ mice [15]. Studies on the 5′-flanking region of the mouse 20α-HSD gene demonstrated that the putative Sp-1 site was critical for the expression of the reporter gene on rat luteinized granulose cells [16]. In rats, the regulatory elements of 20α-HSD (Nur77, Ap-1, CRE, PRLRE, and PRE) are present within a 2.5-kb 5′-flanking region of the gene. In addition, it was shown that the deletion of this promoter at the -289 bp positions significantly decreased the basal promoter activity [17]. PGF_2_α induces the expression of the nuclear orphan receptor and transcription factor Nur77 in the CL, which in turn leads to the stimulation of 20α-HSD, triggering a decrease in serum progesterone, which is essential for parturition [18]. Thus, Nur77 plays an important role in ovarian physiology by mediating the PGF_2_α-mediated induction of 20α-HSD in rats. In the human *20α-HSD* gene, the transcription factor-binding site for NF-Y/CEBP located between -128 and -88 is involved in controlling the basal transcription of 20α-HSD in ovarian, lung, and liver carcinoma cells [19]. The transcription factor Sp-1 regulates the overexpression of 20α-HSD in HT29 human colon cells, which are resistant to methotrexate [20]. The activity of the human *20α-HSD* promoter luciferase was increased by 1.7-fold upon treatment with PGF_2_α and oxytocin on porcine ovarian follicle cells [21].

Among primates, 20α-HSD has been found in the placenta of humans [22], rhesus monkeys [23], and baboons [24]. In humans, the activity of 20α-HSD increased by 5-fold in the placenta from mid to late gestation [25]. The progesterone levels in baboon fetuses declined by almost 6-fold from mid to late gestation and this decrease was accompanied by a substantial increase in the concentration of 20α-OHP, a major metabolite of progesterone [24]. Fetal progesterone concentrations decreased during late pregnancy in the rhesus monkey [26]. In recent reports, the expression of human 20α-HSD was detected in the kidneys [27], adipose cells [28], skin [29], osteoblasts [30], and carcinoma cells from the lung [31], endometrium [32,33], ovaries [34], guts [35] and skin [36]. Over expression of 20α-HSD has been observed in non-small-cell lung cancer [33] and in esophageal and breast cancer [37,38].

We previously showed that 20α-HSD was highly detected in ovarian and placental tissues during the pre-ovulation and pre-parturition phases. It was mainly localized in the syncytiotrophoblast of the placenta and isthmus cells of the oviduct in macaque monkeys [23]. However, the function and regulation of this enzyme in primate reproductive physiology are not well known. Human *20α-HSD* gene is controlled by Sp-1 [19], but there is no report on the regulation of the monkey *20α-HSD* promoter activity. Therefore, we analyzed that the *20α-HSD* gene could be regulated by different transcriptional factors depending on the species.

In the present study, to gain detailed information on the transcriptional regulation factors, we investigated the 5′-flanking region of the monkey *20α-HSD* gene promoter. We demonstrated that the Ap-1 site of the monkey *20α-HSD* gene is essential for its promoter activity, is related to the interaction of the Ap-1 protein with the nuclear extract from Chinese hamster ovary (CHO) K1 cells, and influences the expression of the Ap-1 transcription factor in pre-parturition placenta and CHO-K1 cells.

## Results

### Analysis of putative transcription factor binding sites in the monkey *20α-HSD* gene promoter

**Figure 1:**
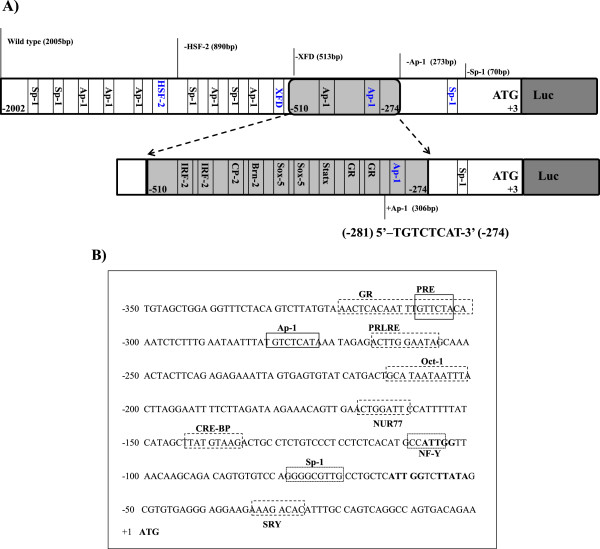
**Putative transcription factor binding sites in the 5′-flanking region of the monkey *20α-HSD *gene. A)** Sequences approximately 2.5 kb upstream from the translation start codon of the monkey 20α-HSD gene. Putative transcription factor binding sites were identified using the TFsearch software. The region includes GR, Ap-1, Oct-1, Sp-1, CRE-BP1 and others. The TATA (-52 → -55) box and two putative CCAAT (-59 → -63; -105 → -109) box sites are indicated in bold. **B)** The translation start codon is indicated as +1. The region between -350 and -53 has a cluster of transcription factor binding sites that are believed to be important for the basal expression of the monkey *20α-HSD* gene.

The monkey *20α-HSD* gene was used to construct the 5′-2005 bp basal promoter region. The transcription factor binding sites of the monkey *20α-HSD* gene promoter was analyzed using the TFsearch software. The promoter region includes a TATA (-52 → -55) box and two putative CCAAT (-59 → -63; -105 → -109) boxes. Moreover, plural transcription factor binding sites were identified, such as Ap-1, Oct-1, Sp-1, NUR77, NF-Y (nuclear factor-Y), CRE-BP (cAMP-response element), SRY, and PRLRE (prolactin response element) (Figure 1A). Various lengths of the 5′-flanking region were constructed to identify the regulatory region crucial for the transcription activity of the *20α-HSD* gene. The deleted mutant promoters were designed as -890-Luc (HSF-2), -510-Luc (XFD), -273-Luc (Ap-1), and -70-Luc (Sp-1), and three mutants were cloned into the PGL-3 Basic vector (Figure 1B).

### Activity of the deleted promoter regions of monkey 20α-HSD

**Figure 2:**
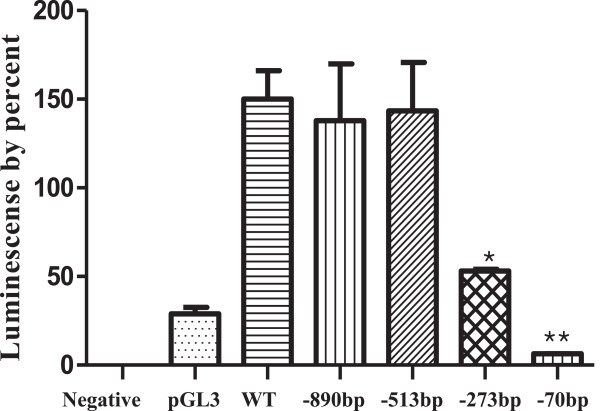
**Promoter activity of the serially deleted 5′-flanking region of the monkey *20α-HSD *gene.** The -890-Luc, -513-luc, -273-Luc, and -70-Luc fragments were generated by PCR amplification and inserted into the pGL3-basic vector. CHO-K1 cells were transfected with the constructs. The results are presented as a percentage of the relative luciferase activity compared with the wild-type. Graphs show the mean ± SEM of three independent experiments.

**Figure 3:**
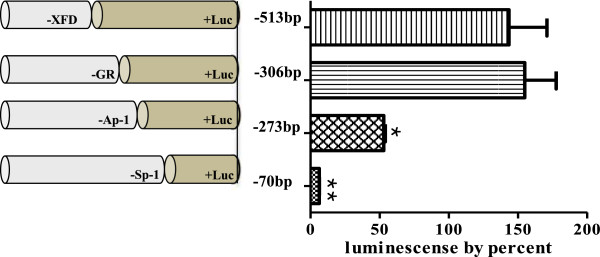
**Promoter activity of the deletion regions with/without the Ap-1 site.** The deletion constructs with/without the Ap-1 site were amplified and then inserted into the pGL3-basic vector. The promoter activities of the constructs were analyzed by transfection into the CHO-K1 cells. The -306-Luc fragment has an Ap-1 site (5′-TGTCTCAT-3′) from -281 to -274. The -273-Luc fragment does not have an Ap-1 site.

**Figure 4:**
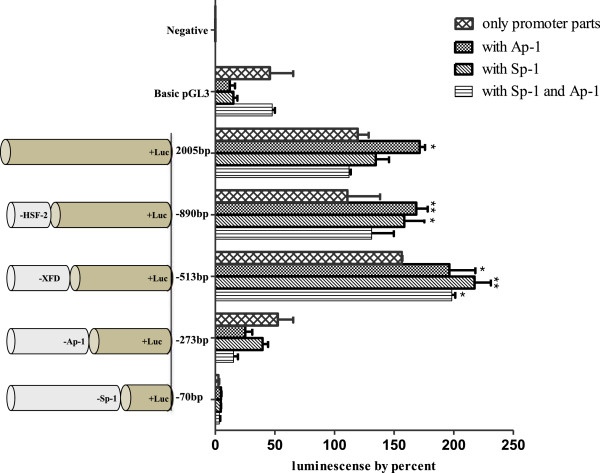
**Transcription activity of the serially deleted promoter regions.** The pGL3-basic vector constructs containing fragments of various lengths were transfected or co-transfected with the pCMV-Sp1 or pSG5-Ap1 expression vectors into CHO cells. For the co-transfection experiments, the luciferase reporter constructs and factor expression vectors were used at 1:4 concentration ratio. The relative luciferase activity (mean ± SEM for triplicate wells) is presented as the percentage of the activity resulting from the promoter transfection alone in each group.

The pGL3-basic vectors were constructed with promoter deletion mutants of different lengths and their activities were assayed in hamster ovarian cells in comparison with the 2005-bp promoter + pGL3 vector (positive control) and non-transfected cells (negative control). As shown in Figure 2, the series of deletion constructs had similar promoter activities. However, when the two constructs -273-Luc and -70-Luc were transfected, the luciferase activity dramatically decreased. In particular, -70-Luc activity was identical to that of the basic pGL3 expression plasmid. Thus, we postulated that the Ap-1 site (-281 to -274) plays an important role in the activation of the promoter of the monkey *20α-HSD* gene. The -306-Luc and -513-Luc constructs containing the Ap-1 site were generated, and their luciferase activities were almost the same (Figure 3). The activity of -273-Luc highly decreased compared to that of -513-Luc and -306-Luc. Next, to investigate whether these factors affected the transcription of monkey *20α-HSD* gene, we transiently co-transfected the constructs into CHO cells in combination with the Ap-1 or Sp-1 expression plasmids individually and together. The luciferase activity of the three constructs, with the exception of -273-Luc and -70-Luc, co-transfected with the Ap-1 and Sp-1 expression plasmids was high compared to that when only one plasmid was transfected. However, the activity of the -273-Luc and -70-Luc constructs, which did not contain the Ap-1 binding site, did not increase when co-transfected with the Ap-1 and Sp-1 expression plasmids (Figure 4). Therefore, the promoter activity of the monkey *20α-HSD* gene may be related to the Ap-1 transcription factor located between -281 and -274. Moreover, the Sp-1 site between -79 and -70 bp is also dispensable to promoter activity (Figures 3 and 4). This region specifically contains IRF-1, 2, Brn-2, CP-2, Sox-5, and GR (Figure 1A). It is also interesting to note that the region between -273 and -70 contains critical regulation sites (e.g., NF-Y, NUR77 and Sp-1; Figure 1B).

### Analyses of the putative Ap-1 binding site by site-directed mutagenesis

**Figure 5:**
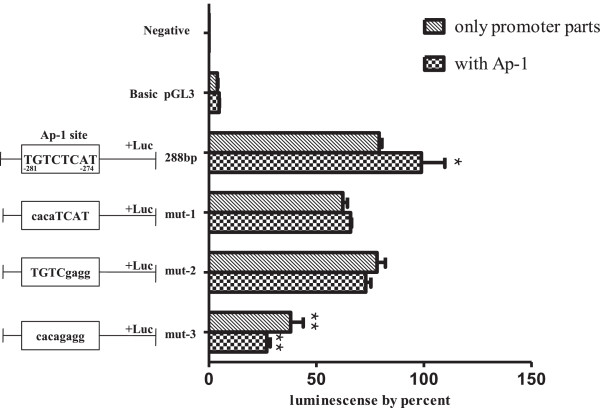
**Effect of nucleotide conversion mutation in the putative Ap-1 site on the promoter region of the monkey *20α-HSD *gene on the transcription activity.** The Ap-1 transcription factor binding sites are indicated in bold and the conversion-mutated nucleotides are indicated by a smaller character. The luciferase reporter constructs and Ap-1 transcription factor expression vectors were co-transfected at 1:4 concentration ratio. The relative luciferase activity (mean ± SEM for triplicate wells) is presented as the percentage of the activity of the 288 bp-pGL3 construct.

Based on the above results, to investigate whether preventing the binding of Ap-1 resulted in decreased the transcription of the *20α-HSD* gene, we constructed the -288-Luc deletion mutant including the Ap-1 binding site (5′-TGTCTCAT-3′). Additionally, we also constructed mutants within the Ap-1 binding site (Table 1), transfected them into CHO cells alone or with the Ap-1 transcription factor, and compared the results with the positive control (-288-Luc). As shown in Figure 5, the luciferase activities of mutant-1 (mut-1) and mutant-2 (mut-2), in which half of the Ap-1 site nucleotides were mutated, were not significantly affected. However, the transcription activity of mutant-3 (mut-3), which had all eight nucleotides of the Ap-1 region mutated, significantly decreased. Moreover, the activity of -288-Luc co-transfected with the Ap-1 expression plasmid increased more than that of mut-1. The activity of mut-2 and mut-3 co-transfected with the Ap-1 expression plasmid did not change. However, as shown in Figure 5, the activity of mut-3 co-transfected with Ap-1 decreased. These results demonstrate that Ap-1 transcription factor (5′-TGTCTCAT-3′) has a specific binding site for the monkey *20α-HSD* gene located in the -281 → -274 region.

**Table 1:** Oligonucleotides used for the construction of the reporter vectors and EMSA

**Constructs**	**Nucleotide sequence (5′ 3′)**
pGL3 (-2005-Luc)	GAG ACG GGG TTT CTC CAT
pGL3 (-890-Luc)	TCT TAC AAG GCT AAT AAG AA
pGL3 (-513-Luc)	AGT AAA CTT TAA TTT TTA AT
pGL3 (-273-Luc)	AAA TAG AGA CTT GGA ATA GC
pGL3 (-306-Luc)	ACA AAT CTC TTT GAA TAA TT
pGL3 (-288-Luc)	TTT ATG TCT CAT AAA TAG AG
pGL3 (-70-Luc)	CTG CTC ATT GGT CTT ATA GC
pGL3 Reverse	CAT TTC TGT CAC TGG CCT
EMSA probe WT	GAA TAA TTT ATG TCT CAT AAA TAG AGA CTT
EMSA probe mt-1	GAA TAA TTT Aca caT CAT A AA TAG AGA CTT
EMSA probe mt-2	GAA TAA TTT ATG TCg agg AAA TAG AGA CTT
EMSA probe mt-3	GAA TAA TTT Aca cag agg AAA TAG AGA CTT

### Ap-1 expression in CHO cells and monkey placenta and analysis by EMSA

**Figure 6:**
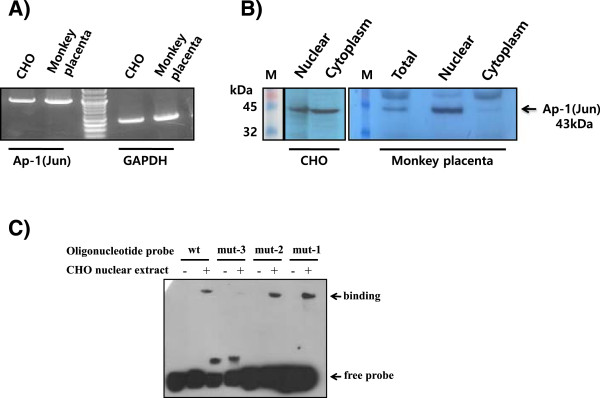
**Expression of the Ap-1 (Jun) factor and EMSA analysis with nuclear extract from CHO-K1 cells. A)** Ap-1 mRNA was detected in CHO cells and monkey placenta by RT-PCR. **B)** Ap-1 protein was analyzed in the total, nuclear, and cytoplasmic proteins extracted from CHO cells by western blot. **C)** EMSA was performed for the wild-type (-288-Luc) and nucleotide-converted mutants (mut-1, mut-2, mut-3) with nuclear extract (3 μg) from CHO cells using 20 fmol of biotin-labeled double-stranded oligonucleotide probe. The protein-DNA complex was transferred to a Zeta probe membrane. Detection of biotin-labeled DNA was performed using the LightShift chemiluminescent EMSA kit and exposed to X-ray film.

The Ap-1 expression level in the placenta from the pre-parturition period and the CHO-K1 cell line was determined by RT-PCR and Western blot (Figure 6A,B). Strong expression of Ap-1 mRNA was detected in both CHO-K1 cells and placenta at pre-parturition. We also found that the Ap-1 protein was coordinately expressed as a nuclear and cytoplasmic protein in CHO cells. However, the expression of the Ap-1 protein in the monkey placenta was weak. Consensus fragments of the oligonucleotide probes for the Ap-1 mutants were also synthesized. For the EMSA, a nuclear protein extract was prepared from CHO cell and used with 3′-end biotin-labeled 30 bp (from -292 nt to -262 nt) oligonucleotide probes. Preliminary EMSA analysis showed the presence of Ap-1 protein-DNA complexes. The mutants used for the EMSA (mut-1, -2, and -3) were similar to those employed in the luciferase assay. The binding of mut-1 and mut-2, which had half of the nucleotides mutated, did not compete with the DNA binding as well as the WT construct. However, mut-3, which had all the nucleotides mutated, did compete with the DNA binding (Figure 6C). Thus, partial mutations of the Ap-1 binding site did not affect the basal activity. Therefore, in the present study, we have functionally characterized the monkey *20α-HSD* gene basal proximal promoter and provided evidence of the role of the Ap-1 in CHO cells.

## Discussion

In the present study, we characterized for the first time the transcription activity and regulation of the 5′-flanking region of the monkey *20α-HSD* gene located in the -281 → -274 region relative to the translational start. This study demonstrated that the Ap-1 site plays a crucial role in the transcription of the monkey 20α-HSD gene in CHO cells. Moreover, we clearly demonstrated that Ap-1 is indispensable for the regulation of the expression of the *20α-HSD* gene. The Ap-1 transcription factor is crucially involved in a multitude of cellular processes, including development and differentiation, cell proliferation, apoptosis, oncogenic transformation, and the response to genotoxic agents [39,40]. The activity of this transcription factor is modulated by growth factors, cytokines, and tumor promoters, such as the phorbol ester 12-O-tetradecanoylphorbol 13-acetate (TPA) [40]. Jun homodimers have less DNA binding activity than do Jun/Fos heterodimers [41]. Moreover, the function of the Ap-1 dimer is cell type-specific and cell cycle-dependent [42,43]. Recently, we reported that the monkey *20α-HSD* mRNA was highly expressed in pre-parturition placenta and pre-ovulation ovary, and it was localized in the syncytiotrophoblast of the placenta. The promoter region of the 5′-flanking region was cloned and the EGFP expression signal under the monkey *20α-HSD* 2005-bp promoter functioned normally in the CHO cell line [23].

In this study, we analyzed the expression of the Ap-1 factor in the pre-parturition placenta of monkey and in hamster ovarian cells. In rat ovarian cells, the Ap-1 factor exhibited a specific expression pattern and response to hormones. Rapid induction of JunB, c-Fos, and Fra-2 by FSH revealed a pattern of other immediate-early genes in granulose cells [44]. Ap-1 proteins are specific in the process of invasion of human maternal tissue by the trophoblast. Most of the Ap-1 family factors are also expressed in the intermediate trophoblast of the placenta during the first trimester and later during pregnancy, with the expression being stronger for JunD and Fra2, followed by c-Jun, c-Fos, and FosB. Furthermore, JunD was expressed in the syncytiotrophoblast [45]. Ap-1 is involved in placenta-specific expression of the mouse and human lactogen I (PL) gene [46,47], human chorionic somatomammotropin A and B [48], human gonadotropin-releasing hormone receptor gene [49], and the collagen digesting enzyme metalloproteinase (MMP)-1 gene that contains an AP-1 response element in its promoter region [50]. In humans, the expression of Ap-1 and NFkB increased in the cervical stroma at the time of parturition. In particular, c-Jun significantly increased in the end of pregnancy and c-Fos immediately increased after parturition, suggesting MAPK activation during cervical ripening before labor [51,52].

In the present study, a reporter assay using constructs of various lengths of the 5′-flanking region revealed that the Ap-1 region between -281 and -274 bp was essential for transcriptional activity. According to the gel shifting assay, the mutant mut-3, which had all the nucleotides mutated, did not have DNA binding activity compared to the wild type, mut-1, or mut-2 Ap-1 factor binding sites. Recent studies demonstrated that human 20α-HSD was regulated by the nuclear factor-Y (NF-Y) in human ovarian, liver, and lung carcinoma cells [19]. However, gel shift assays revealed an increase in Sp1 and Sp2 binding compared to sensitive cells in methotrexate-resistant HT29 human colon cancer cells, without differences in the Sp1 protein levels [20]. A reporter assay of the 5′-flanking region of the mouse *20α-HSD* gene revealed that the region between -83 and 60 bp upstream of the transcription start site was essential for the transcriptional activity in rat luteinizing granulose cells [16]. A putative Sp1 site in this region was critical for the expression of the reporter gene. Finally, the promoter activity was enhanced by the co-transfection of Sp1 expression vector. Thus, the Sp-1 site between -79 and -70 bp needs to be mutated and the activity of the monkey *20α-HSD* mutant promoter tested. However, our results indicate that the monkey *20α-HSD* gene was regulated by Ap-1. Thus, we suggest that the *20α-HSD* gene could be regulated by different transcriptional factors depending on the species.

PRL signals through the Jak2/Stat5 pathway downregulate rat 20α-HSD expression in the decidua. Thus, decidual PRL plays an important role in pregnancy by repressing the expression of interleukin-6 (IL-6) and 20α-HSD in the decidua [53]. The different types of human 20α-HSD inhibitors were reported in an update on the design of potent and active inhibitors based on the crystal structure of the enzyme-inhibitor complex [52]. PGF_2_α induces the expression in the CL of the nuclear orphan receptor and transcription factor NUR77, which in turn leads to the transcriptional stimulation of rat 20α-HSD, triggering the decrease in serum progesterone, which is essential for parturition [7]. The activation of Jun D through a calmodulin-dependent activation of extracellular signal regulated kinases (ERk) 1/2 mediates NUR77 induction by PGF_2_α [18]. When forskolin was used to treat luteal cells transfected with the 2.5-kb rat *20α-HSD* promoter reporter gene, it caused a dose-dependent inhibition of luciferase activity within 24 h [17]. We also collected data on the acceleration and downregulation of the monkey *20α-HSD* gene promoter by PGF_2_α and forskolin (unpublished data). In humans, during cervical ripening and dilation, the presence of the 20α-HSD enzyme results in progesterone inactivation, attenuation of progesterone action, and cervical ripening [54]. In mouse, knockout experiments have revealed non-redundant functions of most of the Ap-1 family members. With regard to the utero-placental system, these experiments pointed to a specific role for JunB and Fra-1. Inactivation of each of the corresponding genes led to severe growth retardation and early fetal death due to impaired vascularization of the decidua [55,56]. Although the NUR77, NF-Y, and Sp1 sites were contained inside the Ap-1 site of the monkey *20α-HSD* promoter, we did not perform analyses to detect the complete suppression of the transcriptional activity of the deletion mutants of the Ap-1 site. However, the *20α-HSD* gene was regulated by these factors in humans, mice, and rats. Thus, further studies aiming to clarify the regulation of the *20α-HSD* gene promoter are needed.

## Conclusions

Taken together, these findings indicate that the Ap-1 transcription factor might play a dynamic role in monkey and mammalian reproductive physiology. Our previous study determined that the 20α-HSD enzyme is important in the monkey estrous cycle and parturition. In this study, we suggest that *20α-HSD* gene expression in the ovary and placenta is regulated by Ap-1 in the monkey reproductive system during pregnancy and pre-parturition. Furthermore, we suggest that the 20α-HSD activity may be important for protecting the fetus from high progesterone levels during parturition because Ap-1 regulates *20α-HSD* gene expression in the placenta and ovaries.

## Methods

### Animal tissues and cell lines

Tissue samples were obtained from the Korea National Primate Research Center (Ohchang, Korea). Placental tissues from an 8-year-old female rhesus monkey of Chinese origins were collected by cesarean section in the pre-parturition period as described previously [23]. The tissues were immediately frozen in liquid nitrogen and stored at -80°C until use. CHO-K1 cells were obtained from the Japanese Cancer Research Resources Bank (Tokyo, Japan). CHO-K1 cells were cultured in Ham's F12 medium (Gibco, MD) containing penicillin (50 U/mL), streptomycin (50 mg/mL), glutamine (2 mM), and 10% fetal bovine serum (FBS). The cells were incubated at 37°C under 5% CO_2_ atmosphere. All animal housing and experiments were performed in accordance with the Korea Research Institute of Bioscience and Biotechnology (KRIBB) Institutional Animal Care and Use Committee Guidance (Accepted No. KRIBB-AFC-09017).

### Construction of luciferase reporter vectors and Ap-1 site mutants

The monkey *20α-HSD* gene promoter (2005-bp) was cloned using the long amplification polymerase chain reaction (LA-PCR) cloning method as described previously [23] and the potential transcription factor binding sites within the *20α-HSD* gene promoter were screened using the TFsearch program (Kyoto University, Japan). To identify the critical trans-activation region of the *20α-HSD* promoter, a series of deletion fragments [-890-Luc, -513-Luc, -306-Luc, -273-Luc, and -70-Luc relative to the translational start codon (+1)] were generated by PCR amplification using the template cloning vector containing the 2005-bp *20α-HSD* promoter region and the primers shown in Table 1. The primers contained the *Kpn*I site at the 5′-end and the *Xho*I site at the 3′-end of each fragment. The PCR fragments were subcloned into the pCR2.1 vector and verified by sequencing to rule out the possibility of any PCR error. The correct plasmids were digested with *Kpn*I and *Xho*I, and then inserted into the same digested sites of the pGL3-Basic luciferase reporter vector (Promega, Madison, WI). Nucleotide inversion mutants (mut-1, mut-2, and mut-3) of the putative Ap-1 site were constructed *according to* the two-step overlapping PCR method [57] using the primers shown in Table 1. PCR was performed with Ex taq polymerase (TaKaRa, Osaka, Japan), and the mutant fragments were subcloned into the pCR 2.1. Finally, the mutants were sequenced and inserted into the pGL3 luciferase vector as described above.

### Transient transfection and luciferase assay

For the luciferase assay, CHO cells were plated at density of 0.5 × 10^5^ cells per well in 500 μL of growth medium without antibiotics 24 h before transfection. On the next day, the cells were washed twice, and 150 μL of Opti-MEM (Invitrogen, Carlsbad, CA) was added to each well. Each vector (0.8 μg) was mixed with 1 μL of Lipofectamine^TM^2000 (Invitrogen, Carlsbad, CA) reagent in 100 μL of Opti-MEM and incubated at room temperature for 20 min. Afterwards, the Lipofectamine-DNA complex was added to each well. After 4 h, 250 μL of CHO growth medium containing 20% (v/v) FBS was added. After incubation for 24 h, the cells were washed twice, 500 μL of CHO-S-SFM-II was added to each well, and the cells were incubated for additional 24 h. Finally, the luciferase activity was measured using a Dual-Glo^TM^ luciferase assay system kit and a 20/20 Glomax Luminometer (Promega, Madison, WI) according to the manufacturer’s instructions. Ap-1 (c-Jun: AF069514) and Sp-1 expression vectors were provided by Dr. K. Imakawa (The University of Tokyo) and Dr. YC. Chang (Catholic University of Daegu School of Medicine). Rabbit polyclonal antibody against mouse Jun was bought from Santa Cruz Biotechnology (Santa Cruz, CA, USA).

In co-transfection experiments, pCMV-Sp1 or pSG5-Ap1 expression vector was mixed with the luciferase vectors at 1:4 concentration ratio. All experiments were repeated at least three times, and representative results were shown. The results of the luciferase assays are expressed as the mean ± standard error of the mean (SEM).

### cDNA synthesis and polymerase chain reaction

Total RNA was extracted using TRIzol reagent (Invitrogen, Carlsbad, CA). Monkey placenta and CHO cells (100 mg) were added to 1 mL of TRIzol reagent and homogenized. Then, 0.2 mL of chloroform was added per 1 mL of TRIzol reagent, and the samples were vigorously vortexed for 15 s. Samples were centrifuged at 12,000 × *g* for 15 min at 4°C. After centrifugation, the upper aqueous phase was transferred into a fresh tube and 0.5 mL of isopropyl alcohol was added. The RNA pellet was washed twice with 75% DEPC water. Finally, the concentration and purity of the RNA was determined by spectrophotometry at 260 nm and 280 nm. cDNA was synthesized as described previously [23]. Two microliters of cDNA was used in each PCR. The Ap-1 (Jun) region was amplified by using forward (5′-ATG ACT GCA AAG ATG GAA ACG-3′) and reverse (5′-TCA AAA TGT TTG CAA CTG CTG CTG-3′) primers. The PCR was performed under the following conditions: 94°C for 1 min; 30 cycles of 94°C for 30 s, 58°C for 30 s, and 72°C for 50 s; final extension at 72°C for 10 min. Primers for glyceraldehyde 3-phosphate dehydrogenase (GAPDH) were used for the normalization of the expression of Ap-1. The sequences of the forward and reverse primers were 5′-ACC ACA GTC CAT GCC ATC AC-3′ and 5′-TCC ACC ACC CTG TTG CTG TA-3′, respectively. The expected length of the PCR fragment was 452 bp. The PCR conditions were 26 cycles of 98°C for 10 s, 55°C for 20 s, and 72°C for 20 s. Gel electrophoresis was used to analyze 5 μL of the PCR products.

### Preparation of nuclear extracts and Western blot analysis

Nuclear extracts were prepared according to the protocol of the NE-PER Nuclear and Cytoplasmic Extraction Reagents Kit (Thermo Scientific, Marietta, OH). For nuclear extraction, the CHO cells were seeded at a density of 1.5 × 10^6^ cells in 100-mm culture dishes. After 100% of the dish area was covered by cells, the cells were trypsinized and washed with ice-cold 1× PBS and centrifuged at 500 × *g* for 5 min. Then, the cell pellet was dried. The cells were re-suspended in 200 μL ice-cold CERI buffer containing 2 μL of a protease inhibitor cocktail, vortexed for 15 s, and incubated on ice for 10 min. Then, 11 μL of ice-cold CERII buffer was added to the tube, the samples were vortexed for 5 s, and incubated on ice for 1 min. The tube was centrifuged for 5 min at 4°C, and the supernatant (cytoplasmic extract) was transferred to a pre-chilled tube. Finally, the insoluble fraction containing the nuclei was suspended in 100 μL of ice-cold NER buffer containing a protease inhibitor cocktail, vortexed for 15 s, and placed on ice for 40 min. The tube was centrifuged at 4°C, 16,000 × g for 10 min, the supernatant (nuclear extract) was recovered to a pre-chilled tube, and the extracts were stored at - 80°C until use. The protein concentration was determined by the Bradford’s colorimetric assay (Bio-Rad, Hercules, CA) using bovine serum albumin (BSA) as the standard [58].

Monkey placenta and CHO cell total proteins were extracted using PRO-PREP (Intron Biotech, Seoul) protein extraction solution. Approximately 15 mg of tissues were minced, and the confluent CHO cells in 100-mm dishes were washed in 1× PBS. The samples were then homogenized in 600 μL of PRO-PREP solution. The cell lysis reaction was induced by incubating the cells for 30 min on ice. Then, the samples were centrifuged at 13,000 rpm at 4°C for 5 min and the supernatant was transferred to a fresh tube. Finally, the protein concentration was measured using the Bradford's method [40]. The protein samples (10 μg) were subjected to SDS-PAGE and transferred to a PVDF membrane using a semidry electro-blotter apparatus for 3 h. Then, the membrane was washed with 1× PBST solution and blocked with 5× skim-milk blocking solution for 1 h. The membrane was probed with a c-Jun rabbit monoclonal antibody (Cell Signaling Technology, Danvers, MA) washed to remove the unbound antibody, and incubated with the secondary antibody goat anti-rabbit horseradish peroxidase (HRP; Abcam, Cambridge, MA), for 30 min. Finally, the membrane was incubated for 5 min with 3 mL of Lumi-Light substrate solution and exposed on X-ray film for 30 s–5 min.

### Electrophoretic mobility shifting assay (EMSA)

Wild-type and mutant probes were amplified as double-stranded oligonucleotides (Table 1) from the -291 to -262 region of the *20α-HSD* promoter. Mutation of the binding consensus site was checked by sequencing, and the fragment was purified by the silica-based Gene Clean II kit (MP Biomedicals, Cleveland, OH) and ammonium acetate DNA precipitation method. All the probes were labeled with biotin using the Biotin 3′-End DNA Labeling kit (Thermo Scientific, Marietta, OH) according to the manufacturer’s instructions. For EMSA, 3 μg of the nuclear extract was utilized for the binding reactions with 20 fmol of biotin-labeled double stranded oligonucleotide probe. The EMSA binding reactions were performed at room temperature for 30 min. in a reaction buffer containing 2.5% glycerol, 5 mM MgCl_2_, 0.05% NP-40, and 50 ng of poly-(dI-dC) for each reaction. The reaction mixtures were loaded on a 6% non-denaturation polyacrylamide gel in pre-chilled 0.5% Tris-EDTA buffer and run at 100 V. The protein-DNA complexes were then transferred to a Zeta probe membrane using the Trans-Blot semi-dry method (Bio Rad, Hercules, CA) and cross-linked using the BLX-254 UV cross-linker. Detection of biotin-labeled DNA was performed using the LightShift chemiluminescent EMSA kit (Thermo Scientific, Marietta, OH), and the membrane was exposed to X-ray film.

### Data and statistical analysis

One-way ANOVA Newman–Keuls Multiple Comparison tests were used to compare the results between samples using GraphPad Prism 5 (GraphPad Software, San Diego, CA). Asterisks indicate significant differences from the control group. (*P < 0.05, **P < 0.01, ***P < 0.001).

## Competing interests

The authors declare that they have no competing interests.

## Authors’ contributions

TN performed the experiments. MHK and DJK performed data analysis. KSM wrote the manuscript. Both authors read and approved the final manuscript.
